# Purchase and use of home healthcare devices for the elderly: a pilot study in Shanghai, China

**DOI:** 10.1186/s12889-020-08757-8

**Published:** 2020-05-04

**Authors:** Duojin Wang, Shiyu Liu, Jing Wu, Qinglian Lin

**Affiliations:** 1 Institute of Rehabilitation Engineering and Technology, University of Shanghai for Science and Technology, Jungong Road 516, Shanghai, 200093 China; 2 Shanghai Engineering Research Center of Assistive Devices, Jungong Road 516, Shanghai, 200093 China; 3 School of Economics & Management, Tongji University, Siping Road 1500, Shanghai, 200092 China; 4 School of Management, Xiamen University, Siming South Road, Xiamen, 361005 China

**Keywords:** Purchase and use, Home healthcare devices, The elderly, Survey

## Abstract

**Background:**

In China, home-based healthcare/rehabilitation has always been advocated by the government and is the most prevalent healthcare pattern. However, there is currently no data on how many each product has been purchased, and it is not clear what factors are associated with their use. The research aims to clarify the current practices and attitudes of the elderly on such matters, and further analyze their influence factors.

**Methods:**

This pilot study consisted of two-round regional survey, conducted from July 25 to August 3, 2015 and July 20 to August 10, 2018 respectively. Both surveys released on-site paper questionnaires and collected after filling out in different communities.

**Results:**

Two hundred forty-four valid questionnaires from 52 communities were collected. Compared with 2015 (30.8%), the number of people who did not purchase home healthcare devices in the same area decreased in 2018 (28.2%). Hemopiezometer (44.3%), glucometer (18.4%), massager (21.3%) and walking devices (19.3%) are the four main types of products that urbanites are most willing to buy. In addition, users’ age group, education level, and income level were significantly correlated with the purchase of certain products.

**Conclusions:**

The types of home healthcare devices purchased by respondents are consistent with the distribution of chronic diseases of urban residents in China. The analysis of product brands also revealed the existing problems and huge growth space of the industry market, which also requires the government to introduce relevant policies and measures to regulate the market and accelerate the development of the industry.

## Background

With the demographic aging and the prevalence of long-term care conditions increases, the global healthcare cost is escalating. Many governments have adopted ongoing series of cost-containment attempts to spend their limited financial resources efficiently so that equitable access to healthcare can be provided [1]. China is a nation with a biggest quantity of the elderly and second largest economy in the world. As with the developed world, China’s healthcare system is also under unprecedented pressure.

As the pressure on the formal healthcare system increased, patients are being released from hospitals and other healthcare facilities that still needing care [2]. Current government policy favors shorter hospital stays by providing more health services in their own homes. It is considered to be less burdensome for the public purse and beneficial for the patients who are able to receive care in their own home [3]. However, home healthcare is an extremely diverse enterprise. Although many studies have shown that the form for well-being, goal attainment and functional status of patients play a positive role, the efficacy of much of the current healthcare that takes place in homes is unknown.

Healthcare is an industry which mushrooms all over the world with an extreme pace [4]. As home healthcare expands, increasing amounts of research are conducting. New developments in assistive technology are likely to make an important contribution to the healthcare of elderly people at home. Many studies suggest that in home healthcare, large amounts of information will be transmitted to and from the home in order to ensure the safety and effectiveness of the services. For instance, vital signs or activity can be monitored through sensor devices, with reminders for people to engage in particular activities [5]. “Smart homes” or robots could serve as healthcare coaches [2]. The Internet of Things (IoT) makes the interconnection of identifiable intelligent objects and medical devices within today’s internet infrastructure and eventually form an intelligent cyber-physical pervasive framework in order to make different in-home healthcare solution [6, 7]. Similar studies like ambient intelligence (AmI) techniques in healthcare have also been used to empower people’s capabilities by means of digital home environments that are sensitive, adaptive, and responsive to human requirements, habits, gestures, and emotions [8, 9].

Blok et al. applied the concept of service modularity to the specific field of healthcare, and presented a modular care and service pattern for independently living elderly [10]. The Deloitte’s research also found that the majority of patients have never tried a virtual healthcare (also known as home telecare) visit, but they generally have an acceptable attitude to such service [11]. Grönvall et al. considered participatory design (PD) activities with patients for homecare devices especially in relation to knowledge about settings and how to reconcile differences in interests [12]. On the situation in China, there has been only a few domestic studies on industry trends of home healthcare devices from a macro-economic perspective so far [13, 14].

In general, most of the current research on home healthcare devices is aimed at the development of new systems/products based on sensors and internet technologies, as well as their practical applications. There are few studies on the actual state (including type of purchase, quantity, and brand, etc.) of such home healthcare devices. Home-based healthcare/rehabilitation has always been advocated by the government and is the most prevalent healthcare pattern in China. However, there is currently no data on how many each product has been purchased, and it is not clear what factors are associated with their use. The lack of survey research of such devices in Chinese health service leads to the shortage of relevant objective data, and this further might result in many problems concerning health regulation formulating, establishment of medical service quality system and the corresponding policies of the government.

Therefore, we have conducted a pilot survey of purchase and use of home healthcare devices mainly for the purpose to clarify the current practices and attitudes of the elderly on such matters, and further analyze their influence factors. These investigations may be helpful to set up the appropriate regulatory and educational system of the policy for home healthcare in China.

## Methods

### Survey design

Shanghai is China’s economic center, where the per capita income, consumption concept and levels of residents are among the highest in the country. The survey on residents’ purchase and use of certain products in the area can basically reflect the urban situation in China. Therefore, we conducted a regional survey of the purchase and use of home healthcare devices targeting the elderly over 60 years old living in Shanghai.

**Fig. 1:**
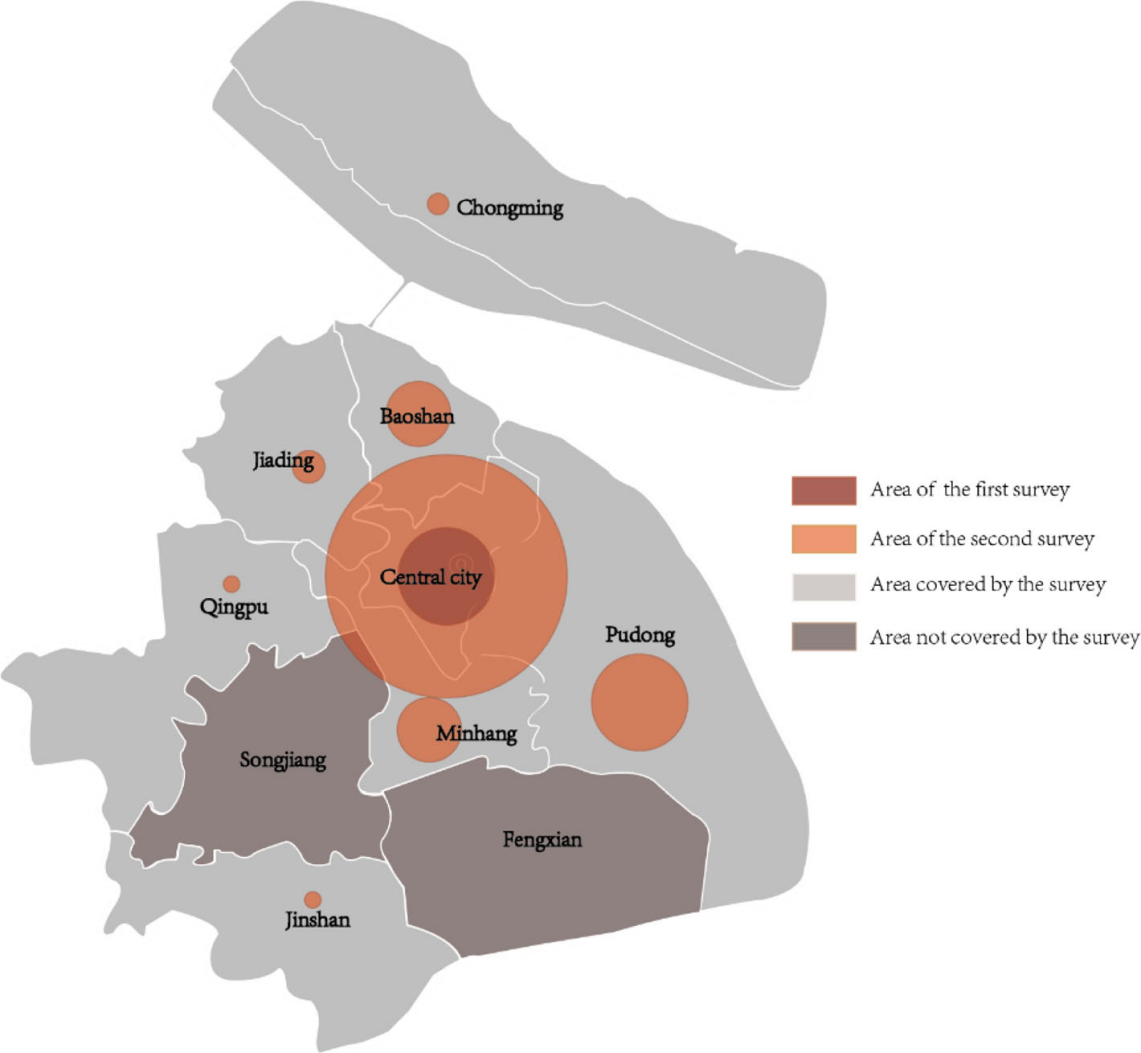
Distribution of responses. NOTE Authors’ drawing based on survey data. In the figure, the size of red dot represents the number of respondents in the corresponding region

This survey has been carried out in two rounds, and both released on-site paper questionnaires. We selected 52 residential communities at random in the target investigation area in advance, and then randomly determined corresponding elderly people over the age of 60 as the respondents based on the size of the community through the residents’ committees.^1^ The background and relevant information of the survey were explained beforehand, and if the elderly agree, they can start to fill in the questionnaires. There is also a description of the survey at the top of each questionnaire. Completion and submission will be considered as the respondent’s informed consent. The first-round survey collected 52 questionnaires in the central city for 10 days from July 25 to August 3, 2015. The second was conducted 3 years after the first survey, from July 20 to August 10, 2018. A total of 200 same questionnaires were collected in 8 of 10 administrative districts for the second time (Fig. 1). All questionnaires were collected on site, and the participation rate of respondents in the two rounds was 100%.

The questionnaire includes 14 items related to the basic data of users, the information of home healthcare devices, and other issues. All questions were closed-ended or partially closed-ended except the basic demographic information of respondents (The questionnaire can be found in Additional file 1).

### Data collection and statistical analysis

We used frequencies, percentages, and graphical display for descriptive analysis. We did a t-test to analyze differences in mean number of the purchase quantity between two rounds of survey. In addition, binary logistic regression was further conducted to clarify how the personal characteristics such as age groups, family status, education and income levels influenced their purchase. We reported odds ratios (ORs) and 95% CIs. Statistical data were entered into a database and processed using SPSS software (Version 20). *P*-Value less than 0.05 was considered as significant.

## Results

As the Fig. 1 shows, the size of colored dot represents the number of respondents in the corresponding area. The central city is the most densely populated region, therefore, through the distribution map, it is shown that this site is the key area in our survey. The specific distribution is shown in the Fig. 1.

The first-round survey only involved the central city, and 52 valid questionnaires were distributed in 10 residential communities (100% valid response rate). The population of Songjiang and Fengxian District marked by brown only accounts for 8% of the city’s population, so the second-round was conducted in 42 residential communities except the 2 districts and 192 valid questionnaires were collected for the 96% valid response rate.

The basic demographic information of the sample is shown as Table 1. The proportion of female (54.9%) in the two rounds was slightly more than male (45.1%). The respondents, average age 74.7 ± 9.3, were assigned to four age groups, which shows a basically uniform distribution. 60 to 69-year-old respondents accounted for around 27.9, 34.4% of total old people investigated were 70 to 79-year-old, 33.6% were 80 to 89-year-old and others account for 4.1%. In addition, the respondents had a wide range of income levels (3682 ± 2078 Yuan). The people with incomes of 2000 to 5999 Yuan accounted for the vast majority of all respondents (77.1%).

**Table 1:** Basic demographic information of the respondents in the survey

Category	Detail	Freq.			Sample No.			Percent (%)			Mean ± SD		
		1st	2nd	T	1st	2nd	T	1st	2nd	T	1st	2nd	T
Gender	M	15	95	110	52	192	244	28.8	49.5	45.1	–	–	–
	F	37	97	134				71.2	50.5	54.9			
Age group (Years)	60–69	13	55	68	52	192	244	25	28.6	27.9	76.9 ± 7.8	74.1 ± 9.6	74.7 ± 9.3
	70–79	20	64	84				38.5	33.3	34.4			
	80–89	19	63	82				36.5	32.8	33.6			
	≥90	0	10	10				0.0	5.2	4.1			
Income level (Yuan)	<2 k	3	28	31	52	192	244	5.8	14.6	12.7	3357 ± 1241	3766 ± 2240	3682 ± 2078
	2 k-3999	39	70	109				75.0	36.5	44.7			
	4 k-5999	7	72	79				13.5	37.5	32.4			
	6 k-7999	3	9	12				5.8	4.7	4.9			
	≥8 k	0	13	13				0.0	6.8	5.3			
Health condition (ICD-10)	Healthy	9	110	119	52	192	244	17.3	57.3	48.8	–	–	–
	HTN	25	39	64				48.1	20.3	26.2			
	DM	9	19	28				17.3	9.9	11.5			
	C00–D48	0	1	1				0.0	0.5	0.4			
	E00-E90	7	2	9				13.5	1.0	3.7			
	G00–G99	0	3	3				0.0	1.6	1.2			
	H00–H59	2	3	5				3.8	1.6	2.0			
	H60–H95	3	0	3				5.8	0.0	1.2			
	I00–I99	10	22	32				19.2	11.5	13.1			
	J00–J99	3	1	4				5.8	0.5	1.6			
	K00–K93	3	5	8				5.8	2.6	3.3			
	M00–M99	13	12	25				25.0	6.3	10.2			
	N00–N99	2	2	4				3.8	1.0	1.6			
Occupati-onal classifyca-tion (OCSM)	MOG A	4	15	19	52	192	244	7.7	7.8	7.8	–	–	–
	MOG B	5	12	17				9.6	6.3	7.0			
	MOG D	5	22	27				9.6	11.5	11.1			
	MOG E	0	2	2				0.0	1.0	0.8			
	MOG F	2	5	7				3.8	2.6	2.9			
	MOG G	0	2	2				0.0	1.0	0.8			
	MOG H	28	96	124				53.8	50.0	50.8			
	MOG K	8	38	46				15.4	19.8	18.9			

NOTE. *T* Total, *M* Male, *F* Female, *k* kilo; *1 k* 1000;

C00–D48 Neoplasms; E00-E90 Endocrine, nutritional and metabolic diseases; G00–G99 Diseases of the nervous system; H00–H59 Diseases of the eye and adnexa; H60–H95 Diseases of the ear and mastoid process; I00–I99 Diseases of the circulatory system; J00–J99 Diseases of the respiratory system; K00–K93 Diseases of the digestive system; M00–M99 Diseases of the musculoskeletal system and connective tissue; N00–N99 Diseases of the genitourinary system

MOG A Professional, Technical and Related Occupations; MOG B Executive, Administrative, and Managerial Occupations; MOG D Administrative Support Occupations, Including Clerical; MOG E Precision Production, Craft, and Repair Occupations; MOG F Machine Operators, Assemblers, and Inspectors; MOG G Transportation and Material Moving Occupations; MOG H Handlers, Equipment Cleaners, Helpers, and Laborers; MOG K Service Occupations, Except Private Household

In terms of health condition, since hypertension (HTN) and diabetes mellitus (DM) are the most common chronic diseases for the elderly in China [15]. Therefore, we listed them separately, and other disease categories were sorted and counted according to the international classification of diseases (ICD-10) [16]. From the survey results, approximately half of the investigators were in good physical condition (48.8%), followed by HTN (26.2%) and diseases of the circulatory system (I00–I99, 13.1%), and more than 10% of those with DM (11.5%) and diseases of the musculoskeletal system and connective tissue (M00–M99, 10.2%).

The previous occupations of respondents according to the Occupational Classification System Manual (OCSM) [17] were divided into eight groups, of which the number of personnel in the Major Occupational Group (MOG) H (50.8%) was far greater than that in the other groups. MOG K and MOG D also accounted for 18.9 and 11.1%, respectively. The proportions of other groups were less than 10%.

### The objective data of respondents on the devices

According to the survey results, the home healthcare devices purchased and used by the respondent were divided into 8 categories: people without any devices (N), hemopiezometer (HM), glucometer (GM), oxygenator (OG), massage devices (MD), protective devices (PD) (including waist supporter and orthosis for temporarily immobilizing), walking devices (WD) (including rollator walker, walking frame and crutch) and wheelchair (WC).

**Fig. 2:**
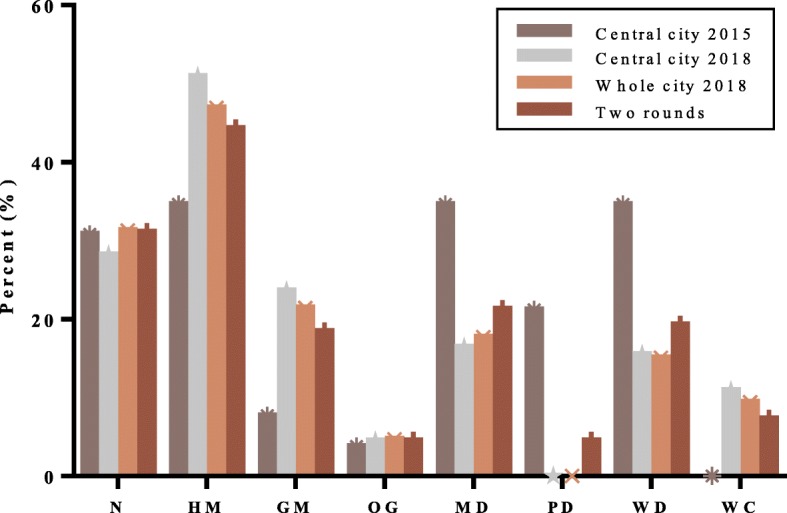
Comparison of the two rounds survey

Figure 2 shows the details of home healthcare devices by respondents in the first-round survey, the central city of the second-round survey, the second-round survey, and the two rounds, respectively. In the first-round, 30.8% of participants did not have any products, followed by the person with the HM (34.6%), MD (34.6%) and WD (34.6%). 21.2% had PD. The proportion of respondents with GM and OG was less than 10%. No one had a WC throughout the first-round survey.

By 2018, the proportion of old people without home healthcare devices in the central city had fallen to 28.2% (31 of 110). The number of owners of HM, GM, OG and WC had increased to varying degrees. Among them, the biggest percentage jump was HM (50.9%), GM (23.6%) and WC (10.9%), and the proportion of OG had only grown slightly (4.5%). The occupancy rates of the devices such as MD (16.4%) and WD (15.5%) were higher than 10%, but they were declining compared with the first-round. In the second-round survey, no one in the central city had protective devices.

As shown in Fig. 2, the result of the whole area was similar as the central city in the second-round. Compared with the first time, we learned that the number of respondents without any home healthcare devices had raised slightly (31.3%) in the entire survey. Home healthcare devices used by the respondents presented by a sequence order of HM (44.3%), MD (21.3%), WD (19.3%), GM (18.4%) and WC (7.3%). OG and PD had the same occupancy rate 4.5%.

### The subjective opinions of respondents on the devices

**Fig. 3:**
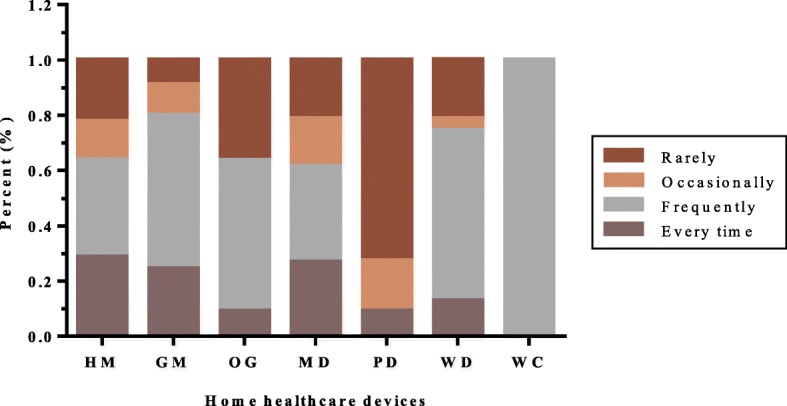
Frequency distribution of each device in the study

**Fig. 4:**
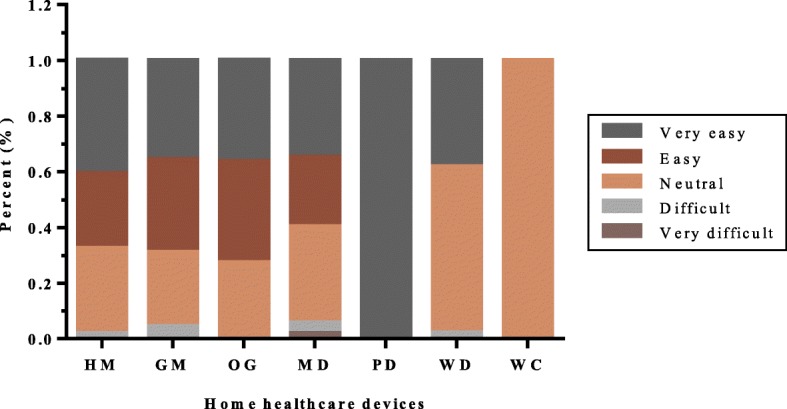
Difficulty distribution of each device in the study

**Fig. 5:**
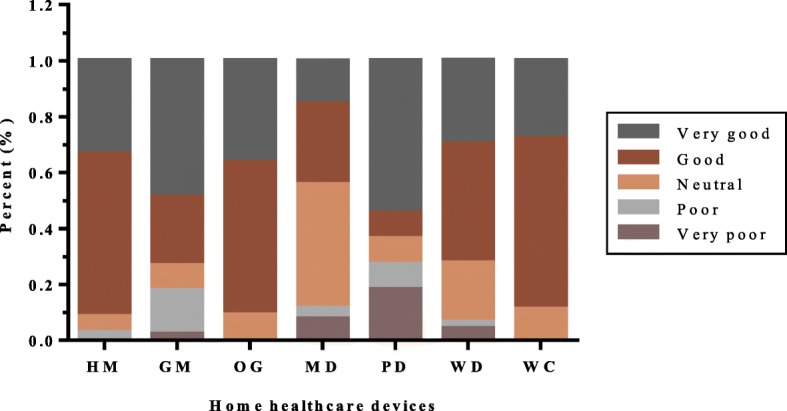
Using effect distribution of each device in the study

We analyzed the subjective opinions of respondents about the home healthcare devices they employed. According to questionnaire responses, Fig. 3, Fig. 4 and Fig. 5 show the use distribution of frequency, difficulty and effect of the eight devices, respectively. As can be seen from the Fig. 3, in addition to the PD, at least 60% of other products were used frequently, some of which were more than 80%. However, only 9.1% of PD were used by people every time.

Users responded differently to the difficulty of using each device. Almost all respondents thought that these devices could be used without barriers, of which the users of PD and WC showed greater consistency. Only a tiny minority said that it is difficult to use the four products such as HM (1.9%), GM (4.4%), MD (5.7%) and WD (2.1%) (Fig. 4).

Figure 5 illustrates the greater diversity of the respondents’ opinions on the using effect. In general, the vast majority were positive (please see the brown and dark gray blocks). However, the effects of five devices had also caused dissatisfaction among some respondents, with more than 10% of uses of GM (17.8%), MD (11.5%) and PD (27.3%). In addition, users who thought that HM and WD were ineffective accounted for 1 and 6% respectively.

### The correlation between basic information of the respondents and purchase/use of the home healthcare devices

Due to the obvious difference in the sample size of the two surveys, we analyzed the difference in the purchase quantity of home healthcare devices for the respondents with three pairs of data-the two rounds, the first-round and the second-round in the central city, the central city and the suburb in the second round, respectively. As shown in Table 2, although the mean number of devices purchased by users was different to some extent, there was no significant statistical difference in each pair of samples. In other words, the purchase of devices in the second survey was similar to that in the first survey, whether it was in the central city or the whole area, and the results were also the same between central and suburban areas in 2018.

**Table 2:** Comparison of purchase quantity of devices in different stages/regions

Range	n (%)	Mean number (SD)	Percentage with purchase quantity of devices				
			0	1	2	3	≥4
All	244 (100%)	1.20 (1.128)	31.1%	36.5%	18.9%	8.2%	5.3%
2015^*^	52 (21.3%)	1.37 (1.358)	30.8%	36.5%	11.5%	7.7%	13.5%
2018^*^	192 (78.7%)	1.16 (1.057)	31.3%	36.5%	20.8%	8.3%	3.1%
CC	162 (100%)	1.25 (1.176)	29.1%	39.5%	16.0%	8.0%	7.4%
2015^†^	52 (32.1%)	1.37 (1.358)	30.8%	36.5%	11.5%	7.7%	13.5%
2018CC^†^	110 (67.9%)	1.20 (1.082)	28.2%	40.9%	18.2%	8.2%	4.5%
2018	192 (100%)	1.16 (1.057)	31.3%	36.5%	20.8%	8.3%	3.1%
CC^‡^	110 (57.3%)	1.20 (1.082)	28.2%	40.9%	18.2%	8.2%	4.5%
S^‡^	82 (42.7%)	1.10 (1.026)	35.4%	30.5%	24.4%	8.5%	1.2%

*CC* Central city, *S* Suburb

^*, †, ‡^ The paired t-test showed no significant differences (*p* > 0.05) between the two groups

We used binary logistic regression to examine associations between respondents’ personal information (including age, education, family status, and income) and the purchase/use of different home healthcare devices for the second-round survey. As shown in Table 3, some characteristics were likely to influence their purchase choices for certain products. Compared with low-income seniors (under 2000 RMB), those highly paid individuals seemed to have better physical functions, which were in turn likely to leads to the purchase of no home healthcare devices (OR = 7.88, *p* = 0.021). The age was strongly associated with the purchase of different devices. For instance, compared with 60–69 years old seniors, the 80 to 89-year-olds were more likely to buy WD (OR = 4.17, *p* = 0.050), people aged 90 and older tended to have HM (OR = 6.65, *p* = 0.040) and WD (OR = 11.03, *p* = 0.021). In addition, the education had an impact on purchase of HM-e.g., people with junior high school, high school and junior college degree had OR of 3.79, 5.88 and 4.02, which are significantly higher than the primary education or below (OR = 1.0). The same situation also occurred in the purchase of WD by people with junior college degrees (OR = 6.19, *p* = 0.029). Other personal information had a certain affection for the product purchases, but the difference was not statistically significant.

**Table 3:** Odds ratios (OR) for purchase of different home healthcare devices by different personal characteristics

	Personal characteristics	OR (95% CI)	p
N			
Income level	Under 2000	1.00	
	4000–5999	6.51 (1.81–23.43)	0.004
	8000 and above	7.88 (1.37–45.49)	0.021
HM			
Age group	60–69	1.00	
	90 or above	6.65 (1.095–40.354)	0.040
Education	Primary education or below	1.00	
	Junior high school degree	3.79 (1.25–11.46)	0.018
	High school degree	5.88 (1.90–18.20)	0.002
	Junior college degree	4.02 (1.10–14.68)	0.035
WD			
Age group	60–69	1.00	
	80–89	4.17 (1.00–17.34)	0.050
	90 or above	11.03 (1.43–85.10)	0.021
Education	Primary education or below	1.00	
	Junior college degree	6.19 (1.21–31.76)	0.029

## Discussion

**Fig. 6:**
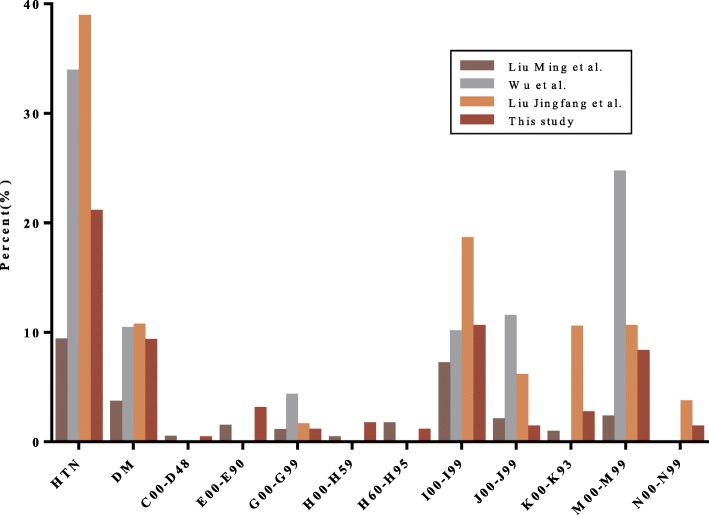
Comparison analysis of survey results

To evaluate the validity of the original data of this survey, comparative analysis was carried out by employing the results of previous research. Liu Ming et al. analyzed the prevalence of major chronic diseases among urban residents in China through a large-scale survey [18]. Wu et al. estimated the best available prevalence for major chronic health conditions among older Chinese adults [19]. Liu Jingfang et al. further teased out the current status and characteristics of chronic diseases in the elderly in China through data of the National Bureau of Statistics [20]. We compared the health conditions of the respondents with the results of the three research according to the ICD-10 classification. Figure 6 illustrates the detailed comparison of disease prevalence rates.

Following the analysis in Fig. 6, it can be clearly seen that HTN, DM, I99-I99 and M00-M99 are the most common types of chronic diseases in urban elderly in China. However, due to the different original samples and conditions, the prevalence of the same disease varies in different studies; e.g. Wu et al. used adults aged 50 years and older in the central and eastern parts of China as the target population for research; Liu Ming et al. only covered the northwest, northeast and southeast cities of China. In addition, time was also an influencing factor; e.g. Liu Jingfang et al. used the official data of the National Bureau of Statistics in 2008. Nevertheless, in general, the chronic disease distribution of the respondents surveyed in this study is similar to others, which also shows the validity of our original data.

The differences between the two rounds in this survey were also analyzed. Compared with 2015, in 2018, the number of respondents who did not purchase any home healthcare devices has dropped in the central city. It can be seen that people are becoming more health-conscious. Moreover, the purchase/use of HM and GM were also on a clear rise in the second-round. This is most likely because the two products are the most accessible to the ordinary people in all categories and are the easiest-to-use healthcare devices.

Essentially, based on the results of Fig. 2 and Fig. 6, it can be found that the respondent’s physical condition is corresponding to the purchased devices. As mentioned before, HTN, DM, I00-i99 and M00-M99 are the four most prevalent diseases in urban elderly in China; HM, GM, MD and WD are exactly four most purchased devices in our survey. People want to control their health information or alleviate the condition by purchasing/using home healthcare devices. For instance, according to the theory of Chinese medicine, massage can accelerate blood circulation. Therefore, many people have used massage devices to try to reduce or eliminate the diseases of the circulatory system (I00-I99).

According to the result of our research, the penetration rate of some home healthcare devices in Shanghai is not much different from that in developed countries. For example, 44.3% of respondents have electronic HM. The rates in the United States and Japan are 50 and 60%, respectively. However, from a national perspective, the penetration rate of HM is only 1.2% [13]. It can be seen that there are great differences between urban and rural areas in China. The situation is similar for other devices.

In 2016, the market size of home healthcare devices in China was approximately 15 billion US dollars, accounting for 27.3% of the entire medical device market [21]. However, the per capita costs of home healthcare devices were anemic. In developed countries, the cost is generally more than 100 US dollars, it is as high as 513 US dollars in Switzerland, while the cost in China is only 6 US dollars [21]. Compared with developed countries, Chinese residents have not yet built consumption habits for home healthcare devices. Therefore, per capita healthcare expenditures are still at a low level. In another point of view, with the aging of the population, the increase in per capita disposable income and the strong propaganda of social media, the public’s awareness of health is constantly enhanced, the demands for various home healthcare devices are gradually expanding. There is a great space to improve the industry market.

The survey results show that some products have a relatively monotonous purchase choice. For example, Omron® holds a large share of the electronic hemopiezometer market, even it is more than 95% in our research. This lack of commercial competition in some areas is not conducive to improvement of user satisfaction and product quality. Moreover, the respondents indicated that some products have different brands and large price gap, but the functions are similar. Under the trend of product homogeneity, the government should roll out corresponding policies to accelerate innovation in the industry, support relevant enterprises in terms of capital, technology and human resources, and introduce a competitive mechanism to improve product diversity. In addition, the government should develop corresponding standards as soon as possible in order to regulate the market and promote the development of the entire industry.

Our research has inevitably some limitations. The relatively small sample size of the first-round survey results in uneven data distribution, there will most likely be bias in the analysis. Therefore, we only conducted correlation and regression analysis for the second-round survey. There are too few samples in some occupational groups, e.g. the responses of MOG E, F and G, so the data collected may have been subjected to career bias. Given the subjective survey data provided by the respondents, there may be a certain level of distortion in the results. Our research aims to reflect the national situation through research in the Shanghai area. However, due to the uneven development of the eastern and western regions of China, and there are also significant differences between the north and the south. Therefore, the results of this study could more accurately reflect the status quo of developed regions in China. These issues are expected to be carried out in our future studies.

## Conclusion

This research indicated that the health awareness of urban residents is constantly increasing in China. The proportion of purchase and use of home healthcare devices is gradually improving. Hemopiezometer, glucometer, massager and walking devices are the four main types of products that urbanites are most willing to buy. Through comparison with the existing studies, it is found that this is consistent with the distribution of chronic diseases of urban residents in China. We also discovered that users’ age group, education, and income level were significantly correlated with the purchase of certain products. In addition, the analysis of product brands also revealed the existing problems and huge growth space of the industry market, which also requires the government to introduce relevant policies and measures to regulate the market and accelerate the development of the industry.

## Supplementary information

**Additional file 1.** Questionnaire about purchase and use of home healthcare devices for the elderly.

## Data Availability

All data and materials related to the study can be obtained through contacting the first author at duojin.wang@usst.edu.cn.

## Abbreviations

ORs: Odds Ratios
HTN: Hypertension
DM: Diabetes Mellitus
ICD: International Classification of Diseases
OCSM: Occupational Classification System Manual
MOG: Major Occupational Group
N: People without any devices
HM: Hemopiezometer
GM: Glucometer
OG: Oxygenator
MD: Massage Devices
PD: Protective Devices
WD: Walking Devices
WC: Wheelchair

## Footnotes

1 In China, a residential community, also called residential quarter, is an urban residential area and its residents administrated by a subdistrict. Every community has a residents’ committee and every committee administers the dwellers living in that community.
